# Supplementary material for: The Severity of COVID-19 Affects the Plasma Soluble Levels of the Immune Checkpoint HLA-G Molecule
Source: Int J Mol Sci. 2022 Aug 27;23(17):9736. doi: 10.3390/ijms23179736 (PMC9456149; doi:10.3390/ijms23179736)
Supplement: Supplementary file 1 [file ijms-23-09736-s001.zip › ijms-1830942-supplementary.pdf]

Supplementary Materials

Original article: "Severity of covid-19 affects the plasma soluble levels of the immune checkpoint HLA-G molecule"

Supplementary Table S1. Characterization of participants according to signs and symptoms of covid-19.

Participant's Classification	Symptoms, Signs, and Parameters
**Healthy Controls**	- Negative for SARS-Cov-2 nucleic acid<br>- No clinical signs
**Mild**	- Positive for SARS-Cov-2 nucleic acid and/or serological test<br>- With or without the following symptoms: diarrhea, cough, fever, headache, loss of taste (ageusia) / smell (anosmia), myalgia, nausea, and vomiting<br>- Oxygen saturation 94-99 % on room air
**Moderate**	- Positive for SARS-Cov-2 nucleic acid and/or serological test<br>- Manifestation of mild disease symptoms including dyspnea<br>- Oxygen saturation ≥ 93% on room air and PaO2/FiO2 250-300 mmHg<br>- Do not need invasive ventilation: nasal catheter (oxygen 2-4 L/min) or oxygen reservoir (oxygen 4-12 L/min)
**Severe**	- Positive for SARS-Cov-2 nucleic acid and/or serological test<br>- Possible admission to intensive care units<br>- Severe respiratory distress<br>- Oxygen saturation < 93 % on room air and PaO2/FiO2 < 250 mmHg<br>- Need non-invasive ventilation: oxygen reservoir or non-rebreathing face mask (oxygen 10-15 L/min)
**Critical**	- Positive for SARS-Cov-2 nucleic acid and/or serological test<br>- Admission to intensive care units<br>- Acute respiratory distress syndrome<br>- Need invasive ventilation<br>- PaO2/FiO2 < 200 mmHg<br>- With or without one or more additional parameters: need hemodialysis, sepsis, septic shock, and multiorgan dysfunction

The participants were classified into five clinical groups, established by symptoms severity, clinical parameters, patient management, and laboratory findings, following the WHO recommendations) [1–8]. Abbreviations: FiO2: fraction of inspired oxygen; PaO2: partial pressure of oxygen.

Supplementary Table S2. Information about r and *p*-values of the correlation matrix shown in Figure 5.

Comparison	Row	Column	Correlation	*p-Value*
1	BMI	Age	0,1229	0,062855932
2	Infection days	Age	0,3824	1,49738E-07
3	Days hospitalized	Age	0,6205	7,93271E-27
4	Clinical Score	Age	0,6486	6,46492E-30
5	O_2 Infused	Age	0,6685	8,43837E-32
6	INR	Age	0,0087	0,902143302
7	Total Leukocytes	Age	0,2334	0,00027325
8	Neutrophil	Age	0,5899	8,43501E-24
9	Lymphocyte	Age	-0,6383	9,48496E-29
10	NLR	Age	0,6103	1,39724E-25
11	Glycemia	Age	0,5144	1,6348E-15
12	C-reactive protein	Age	0,0326	0,765920878
13	IL-12	Age	0,0422	0,520128895
14	TNF	Age	0,0732	0,263718758
15	IL-10	Age	0,5077	8,41774E-17
16	IL-6	Age	0,6357	5,23447E-28
17	IL-1β	Age	0,1251	0,055426557
18	IL-8	Age	0,6660	2,43062E-30
19	sHLA-G	Age	0,2615	4,24493E-05
20	Infection days	BMI	0,0867	0,259296489
21	Days hospitalized	BMI	0,1950	0,002972993
22	Clinical score	BMI	0,2937	5,89609E-06
23	O_2 Infused	BMI	0,2307	0,00045931
24	INR	BMI	-0,1970	0,005647958
25	Total leukocytes	BMI	0,0986	0,136112741
26	Neutrophil	BMI	0,0958	0,147436719
27	Lymphocyte	BMI	-0,0964	0,144882031
28	NLR	BMI	0,1014	0,126970629
29	Glycemia	BMI	0,2232	0,001367423
30	C-reactive protein	BMI	0,0052	0,963785371
31	IL-12	BMI	-0,0510	0,442356983
32	TNF	BMI	0,0133	0,841927868
33	IL-10	BMI	0,1473	0,025766699
34	IL-6	BMI	0,2660	4,5779E-05
35	IL-1β	BMI	-0,0518	0,435542815
36	IL-8	BMI	0,2537	0,000137172
37	sHLA-G	BMI	0,1457	0,027120788

38	Days hospitalized	Infection days	0,4269	3,11116E-09
39	Clinical score	Infection days	0,4186	6,70519E-09
40	O_2 Infused	Infection days	0,4437	7,7815E-10
41	INR	Infection days	-0,1388	0,094785377
42	Total leukocytes	Infection days	0,1437	0,056398953
43	Neutrophil	Infection days	0,3700	4,02054E-07
44	Lymphocyte	Infection days	-0,3960	4,87316E-08
45	NLR	Infection days	0,3955	6,06602E-08
46	Glycemia	Infection days	0,3202	4,58944E-05
47	C-reactive protein	Infection days	-0,0312	0,790647466
48	IL-12	Infection days	0,0470	0,539465841
49	TNF	Infection days	0,0315	0,681237279
50	IL-10	Infection days	0,3019	5,40977E-05
51	IL-6	Infection days	0,3915	9,98929E-08
52	IL-1β	Infection days	-0,0360	0,638578874
53	IL-8	Infection days	0,4385	3,07714E-09
54	sHLA-G	Infection days	0,0486	0,520906168
55	Clinical score	Days hospitalized	0,8284	1,37099E-61
56	O_2 Infused	Days hospitalized	0,8899	2,09201E-81
57	INR	Days hospitalized	-0,1148	0,102931162
58	Total leukocytes	Days hospitalized	0,3299	1,79243E-07
59	Neutrophil	Days hospitalized	0,7284	8,48898E-41
60	Lymphocyte	Days hospitalized	-0,7546	2,99013E-45
61	NLR	Days hospitalized	0,7416	1,27993E-42
62	Glycemia	Days hospitalized	0,5581	1,64134E-18
63	C-reactive protein	Days hospitalized	0,1744	0,108307138
64	IL-12	Days hospitalized	0,0862	0,187684382
65	TNF	Days hospitalized	0,1065	0,103261363
66	IL-10	Days hospitalized	0,6209	1,90259E-26
67	IL-6	Days hospitalized	0,7252	1,21913E-39
68	IL-1β	Days hospitalized	0,1462	0,025026763
69	IL-8	Days hospitalized	0,6795	5,86835E-32
70	sHLA-G	Days hospitalized	0,2289	0,000359545
71	O_2 Infused	Clinical Score	0,8958	5,20703E-84
72	INR	Clinical Score	-0,1057	0,133381204
73	Total leukocytes	Clinical Score	0,3488	3,04232E-08
74	Neutrophil	Clinical Score	0,7023	7,75194E-37
75	Lymphocyte	Clinical Score	-0,7328	1,6728E-41
76	NLR	Clinical Score	0,7206	3,0273E-39
77	Glycemia	Clinical Score	0,6019	5,50832E-22

78	C-reactive protein	Clinical Score	0,3417	0,001284096
79	IL-12	Clinical Score	0,0848	0,195031019
80	TNF	Clinical Score	0,0916	0,161389944
81	IL-10	Clinical Score	0,6189	3,1064E-26
82	IL-6	Clinical Score	0,7849	2,44276E-50
83	IL-1β	Clinical Score	0,1547	0,017625184
84	IL-8	Clinical Score	0,7508	3,21646E-42
85	sHLA-G	Clinical Score	0,3801	1,24206E-09
86	INR	O_2 Infused	-0,0692	0,330157046
87	Total leukocytes	O_2 Infused	0,3101	1,24549E-06
88	Neutrophil	O_2 Infused	0,7263	8,4057E-40
89	Lymphocyte	O_2 Infused	-0,7658	1,40816E-46
90	NLR	O_2 Infused	0,7526	8,44528E-44
91	Glycemia	O_2 Infused	0,5628	8,88037E-19
92	C-reactive protein	O_2 Infused	0,5057	1,26246E-06
93	IL-12	O_2 Infused	0,1148	0,081620195
94	TNF	O_2 Infused	0,1296	0,049139116
95	IL-10	O_2 Infused	0,6146	2,18168E-25
96	IL-6	O_2 Infused	0,8028	2,51121E-53
97	IL-1β	O_2 Infused	0,1487	0,023807786
98	IL-8	O_2 Infused	0,7312	1,43461E-38
99	sHLA-G	O_2 Infused	0,2659	3,65033E-05
100	Total leukocytes	INR	0,1034	0,142061447
101	Neutrophil	INR	-0,0610	0,387615727
102	Lymphocyte	INR	0,0581	0,410446198
103	NLR	INR	-0,0878	0,215414212
104	Glycemia	INR	-0,1535	0,036456594
105	C-reactive protein	INR	0,4793	6,14488E-05
106	IL-12	INR	0,0426	0,548836851
107	TNF	INR	-0,0800	0,260134709
108	IL-10	INR	-0,0660	0,35342934
109	IL-6	INR	-0,0177	0,803868742
110	IL-1β	INR	0,0648	0,362197673
111	IL-8	INR	-0,0116	0,872812168
112	sHLA-G	INR	0,0475	0,500543622
113	Neutrophil	Total Leukocytes	0,4468	3,95787E-13
114	Lymphocyte	Total Leukocytes	-0,4811	2,99472E-15
115	NLR	Total Leukocytes	0,4461	5,44968E-13
116	Glycemia	Total Leukocytes	0,2892	2,1734E-05

117	C-reactive protein	Total Leukocytes	0,1371	0,208093877
118	IL-12	Total Leukocytes	-0,0709	0,279050243
119	TNF	Total Leukocytes	0,1482	0,023069673
120	IL-10	Total Leukocytes	0,2205	0,000662591
121	IL-6	Total Leukocytes	0,2885	6,95599E-06
122	IL-1β	Total Leukocytes	0,1514	0,020268537
123	IL-8	Total Leukocytes	0,2319	0,000440509
124	sHLA-G	Total Leukocytes	0,1444	0,02563749
125	Lymphocyte	Neutrophil	-0,9642	1,2811E-138
126	NLR	Neutrophil	0,9549	5,9644E-126
127	Glycemia	Neutrophil	0,4890	5,81368E-14
128	C-reactive protein	Neutrophil	0,1643	0,130641111
129	IL-12	Neutrophil	-0,0106	0,87218733
130	TNF	Neutrophil	0,1251	0,055454834
131	IL-10	Neutrophil	0,5767	3,11323E-22
132	IL-6	Neutrophil	0,6314	1,51659E-27
133	IL-1β	Neutrophil	0,1265	0,052814858
134	IL-8	Neutrophil	0,5813	8,01876E-22
135	sHLA-G	Neutrophil	0,1661	0,010120135
136	NLR	Lymphocyte	-0,9738	3,4336E-153
137	Glycemia	Lymphocyte	-0,5139	1,75934E-15
138	C-reactive protein	Lymphocyte	-0,2386	0,026972849
139	IL-12	Lymphocyte	-0,0071	0,913278078
140	TNF	Lymphocyte	-0,1157	0,076753975
141	IL-10	Lymphocyte	-0,5943	7,90334E-24
142	IL-6	Lymphocyte	-0,6721	3,00596E-32
143	IL-1β	Lymphocyte	-0,1189	0,068788659
144	IL-8	Lymphocyte	-0,6196	2,37188E-25
145	sHLA-G	Lymphocyte	-0,1661	0,01011719
146	Glycemia	NLR	0,5149	2,07594E-15
147	C-reactive protein	NLR	0,2148	0,048397943
148	IL-12	NLR	0,0055	0,933194961
149	TNF	NLR	0,0840	0,201277701
150	IL-10	NLR	0,5791	2,87651E-22
151	IL-6	NLR	0,6580	2,74221E-30
152	IL-1β	NLR	0,1062	0,105777999
153	IL-8	NLR	0,6037	1,24603E-23
154	sHLA-G	NLR	0,1654	0,010738278
155	C-reactive protein	Glycemia	-0,0877	0,501403057
156	IL-12	Glycemia	-0,0365	0,60238259
157	TNF	Glycemia	0,0138	0,843661268

158	IL-10	Glycemia	0,4252	1,89194E-10
159	IL-6	Glycemia	0,4920	5,86556E-14
160	IL-1β	Glycemia	0,0421	0,547920542
161	IL-8	Glycemia	0,5279	9,50264E-16
162	sHLA-G	Glycemia	0,2593	0,00014993
163	IL-12	C-reactive protein	0,1368	0,217327087
164	TNF	C-reactive protein	0,1449	0,19135936
165	IL-10	C-reactive protein	0,0860	0,439439536
166	IL-6	C-reactive protein	0,3899	0,000269159
167	IL-1β	C-reactive protein	0,1651	0,135706968
168	IL-8	C-reactive protein	0,2082	0,071061249
169	sHLA-G	C-reactive protein	0,1884	0,082334118
170	TNF	IL-12	0,3761	2,61016E-09
171	IL-10	IL-12	0,1145	0,079962887
172	IL-6	IL-12	0,0619	0,34452535
173	IL-1β	IL-12	0,3053	1,84856E-06
174	IL-8	IL-12	0,0364	0,585993827
175	sHLA-G	IL-12	-0,0168	0,798041195
176	IL-10	TNF	0,1921	0,003116923
177	IL-6	TNF	0,0582	0,374677003
178	IL-1β	TNF	0,3315	1,95846E-07
179	IL-8	TNF	0,0446	0,504472816
180	sHLA-G	TNF	-0,1207	0,064617776
181	IL-6	IL-10	0,6873	3,35789E-34
182	IL-1β	IL-10	0,1239	0,057910603
183	IL-8	IL-10	0,7639	1,72124E-44
184	sHLA-G	IL-10	0,2825	1,09839E-05
185	IL-1β	IL-6	0,1872	0,003986332
186	IL-8	IL-6	0,7755	1,25921E-46
187	sHLA-G	IL-6	0,3048	1,91881E-06
188	IL-8	IL-1β	0,0862	0,196647333
189	sHLA-G	IL-1β	0,0202	0,758516596
190	sHLA-G	IL-8	0,3633	1,85843E-08

Supplementary Table S3. Binomial logistic regression coefficient model under collinearity * for severity and outcome of mortality in the study.

Predictor	Estimate	SE	Z	*p*	*Odds ratio*	95% Confidence Interval		VIF	Tolerance
						Lower	Upper		
**Severity**									
Intercept	-5.97374	0.96339	-6.201	< .001	0.00254	3.85e-4	0.0168	-	-
sHLA-G	9.56e-4	0.00287	0.333	0.739	1.00096	0.995	1.0066	1.04	0.959
NLR	0.09505	0.05408	1.758	0.079	1.09972	0.989	1.2227	1.32	0.757
Days hospitalized	0.15963	0.04505	3.544	< .001	1.17308	1.074	1.2814	1.23	0.812
Glycemia	0.00910	0.00374	2.434	0.015	1.00914	1.002	1.0166	1.17	0.852
Comorbidities (present vs absent)	0.74200	0.52025	1.426	0.154	2.10013	0.758	5.8222	1.21	0.828
Sex (male vs female)	0.06467	0.48519	0.133	0.894	1.06680	0.412	2.7611	1.09	0.919
Age, over 60 years	0.04169	0.01465	2.847	0.004	1.04257	1.013	1.0729	1.21	0.823
**Outcome of Mortality**									
Intercept	-7.48800	1.19692	-6.256	< .001	5.60e-4	5.36e-5	0.00585	-	-
sHLA-G	0.00585	0.00241	2.427	0.015	1.006	1.001	1.01062	1.03	0.966
NLR	0.01114	0.01699	0.656	0.512	1.011	0.978	1.04545	1.12	0.890
Days hospitalized	0.13411	0.03546	3.782	< .001	1.144	1.067	1.22583	1.13	0.887
Glycemia	-0.00106	0.00392	-0.271	0.786	0.999	0.991	1.00665	1.22	0.820
Comorbidities (present vs absent)	-0.36565	0.58462	-0.625	0.532	0.694	0.221	2.18188	1.31	0.764
Sex (male vs female)	0.53415	0.49318	1.083	0.279	1.706	0.649	4.48511	1.05	0.956
Age, over 60 years	0.08135	0.01671	4.869	< .001	1.085	1.050	1.12086	1.22	0.821

vs: versus; RNL: neutrophil–lymphocyte ratio.

References

1. Grasselli, G.; Zangrillo, A.; Zanella, A.; Antonelli, M.; Cabrini, L.; Castelli, A.; Cereda, D.; Coluccello, A.; Foti, G.; Fumagalli, R.; et al. Baseline Characteristics and Outcomes of 1591 Patients Infected with SARS-CoV-2 Admitted to ICUs of the Lombardy Region, Italy. *JAMA - Journal of the American Medical Association* **2020**, *323*, 1574–1581, doi:10.1001/jama.2020.5394.
2. Hadjadj, J.; Yatim, N.; Barnabei, L.; Corneau, A.; Boussier, J.; Smith, N.; Péré, H.; Charbit, B.; Bondet, V.; Chenevier-Gobeaux, C.; et al. Impaired Type I Interferon Activity and Inflammatory Responses in Severe COVID-19 Patients. *Science (1979)* **2020**, *369*, doi:10.1126/science.abc6027.
3. Marshall, J.C.; Murthy, S.; Diaz, J.; Adhikari, N.; Angus, D.C.; Arabi, Y.M.; Baillie, K.; Bauer, M.; Berry, S.; Blackwood, B.; et al. A Minimal Common Outcome Measure Set for COVID-19 Clinical Research. *The Lancet Infectious Diseases* 2020, *20*.
4. Xu, X.W.; Wu, X.X.; Jiang, X.G.; Xu, K.J.; Ying, L.J.; Ma, C.L.; Li, S.B.; Wang, H.Y.; Zhang, S.; Gao, H.N.; et al. Clinical Findings in a Group of Patients Infected with the 2019 Novel Coronavirus (SARS-Cov-2) Outside of Wuhan, China: Retrospective Case Series. *The BMJ* **2020**, *368*, doi:10.1136/bmj.m606.
5. Ye, G.; Pan, Z.; Pan, Y.; Deng, Q.; Chen, L.; Li, J.; Li, Y.; Wang, X. Clinical Characteristics of Severe Acute Respiratory Syndrome Coronavirus 2 Reactivation. *Journal of Infection* **2020**, *80*, doi:10.1016/j.jinf.2020.03.001.
6. Office, W.H.O.E.M.R. Updated Clinical Management Guideline for COVID-19. *Weekly Epidemiology Monitor* 2020, *13*.
7. Wei, P.-F. Diagnosis and Treatment Protocol for Novel Coronavirus Pneumonia (Trial Version 7). *Chinese Medical Journal* **2020**, *133*, 1087–1095, doi:10.1097/CM9.0000000000000819.
8. Wan, S.; Xiang, Y.; Fang, W.; Zheng, Y.; Li, B.; Hu, Y.; Lang, C.; Huang, D.; Sun, Q.; Xiong, Y.; et al. Clinical Features and Treatment of COVID-19 Patients in Northeast Chongqing. *Journal of Medical Virology* **2020**, *92*, 797–806, doi:10.1002/jmv.25783.
